# Chinese Herbal Medicine in the Treatment of Depression in Parkinson’s Disease: From Molecules to Systems

**DOI:** 10.3389/fphar.2022.879459

**Published:** 2022-04-13

**Authors:** Yi Zhang, Xiaoman Xu

**Affiliations:** ^1^ Department of Gerontology and Geriatrics, Shengjing Hospital of China Medical University, Shenyang, China; ^2^ Department of Pulmonary and Critical Care Medicine, Shengjing Hospital of China Medical University, Shenyang, China

**Keywords:** active ingredient, depression, neuropharmacology, Parkinson’s disease, Traditional Chinese Medicine

## Abstract

Depression is one of the most common non-motor symptoms in patients with Parkinson’s disease (PD). Depression in PD (DPD) increases the disability rate and reduces the quality of life of PD patients and increases the caregiver burden. Although previous studies have explained the relationship between depression and PD through a variety of pathological mechanisms, whether depression is a precursor or an independent risk factor for PD remains unclear. Additionally, increasing evidence shows that conventional anti-PD drug therapy is not ideal for DPD. Chinese Herbal Medicine (CHM) prescriptions exhibit the characteristics of multi-target, multi-pathway, and multi-level treatment of DPD and may simultaneously improve the motor symptoms of PD patients through multiple mechanisms. However, the specific pharmacological mechanisms of these CHM prescriptions remain unelucidated. Here, we investigated the mechanisms of action of the active ingredients of single herbs predominantly used in CHM prescriptions for depression as well as the therapeutic effect of CHM prescriptions on DPD. This review may facilitate the design of new selective and effective treatment strategies for DPD.

## Introduction

Parkinson’s disease (PD) is the most common neurodegenerative disease, affecting more than 610 million patients worldwide in 2018 (Global, 2018). The clinical symptoms of PD are divided into motor (MS) and non-motor symptoms (NMS) (Tolosa et al., 2006). Bradykinesia, static tremor, muscle rigidity, and postural balance disorders are the four core MS in PD (Brigo et al., 2014). NMS may manifest as olfaction, sleep, and mood disorders and have a greater impact on the quality of life of PD patients than MS (Schapira et al., 2017). Depression in PD (DPD) is one of the most common NMS. However, owing to different diagnostic criteria and observation groups for depression, the prevalence of DPD is uncertain (Dobkin et al., 2020). The prevalence of DPD fluctuates from 4% to 90% (Leentjens, 2004), and that of major depression ranges from 40% to 50% (Reijnders et al., 2008). Patients with DPD may require earlier initiation of dopamine (DA) therapy, have more severe motor dysfunction, faster cognitive decline, reduced quality of life, and increased caregiver burden (Even and Weintraub, 2012; Qian et al., 2017).

Depressive disorder is a group of symptomatic syndromes characterized by significant depressive mood (Schrag and Taddei, 2017). The clinical characteristics of DPD differ from those of primary depressive disorders. DPD is characterized by indifference, lethargy, attention difficulty, reduced euphoria, anxiety, and irritability and includes typical suicidal thoughts without suicidal actions (Sagarwala and Nasrallah, 2020). Self-blame and frustration are more severe in DPD than in primary depression (Marsh, 2013). DPD may be the result of pathological changes, a reaction to PD-related disability, a separate manifestation, or a combination of the three (Schapira et al., 2017). Previous studies on the pathophysiological mechanism of DPD suggest that it shares many mechanisms with depression: First, DPD is significantly associated with dopaminergic denervation of the cingulate gyrus (Frosini et al., 2015), and the destruction of the dopaminergic system leads to the reduction in the activity of the serotonergic system and thus to the expression of depressive behaviors (Lee et al., 2015). Second, the inflammatory response in PD patients is correlated with the occurrence of depression (Pessoa Rocha et al., 2014). Third, changes in serotonergic neurotransmission in PD are associated with the pathogenesis of DPD (Shannak et al., 1994). This mechanism forms the basis for the current clinical application of traditional antidepressants, such as serotonin (5-HT)-reuptake inhibitors, tricyclic antidepressants, and 5-HT/norepinephrine (NE)-reuptake inhibitors, in the treatment of DPD.

Therefore, multiple pathogenic mechanisms may be involved in the pathogenesis of DPD, which may be one of the reasons why a single antidepressant therapy cannot achieve satisfactory therapeutic effects in DPD patients (Seppi et al., 2011). Multi-components contribute to the characteristics of multi-target, multi-pathway, and multi-level of CHM prescriptions in treatment of diseases. With the in-depth research on the pharmacological mechanism of Traditional Chinese Medicine (TCM), CHM prescriptions have been widely used in clinical practice. However, there is still a lack of unified conclusions on the treatment of DPD by CHM prescriptions. Here, we investigated the mechanisms of action of the active ingredients of single herbs predominantly used in CHM prescriptions for depression as well as the therapeutic effect of CHM prescriptions on DPD. This review may facilitate the design of new selective and effective treatment strategies for DPD.

## Relationship Between Depression and Parkinson’s Disease: Prodromal Symptom or Separate Disorder?

**FIGURE 1:**
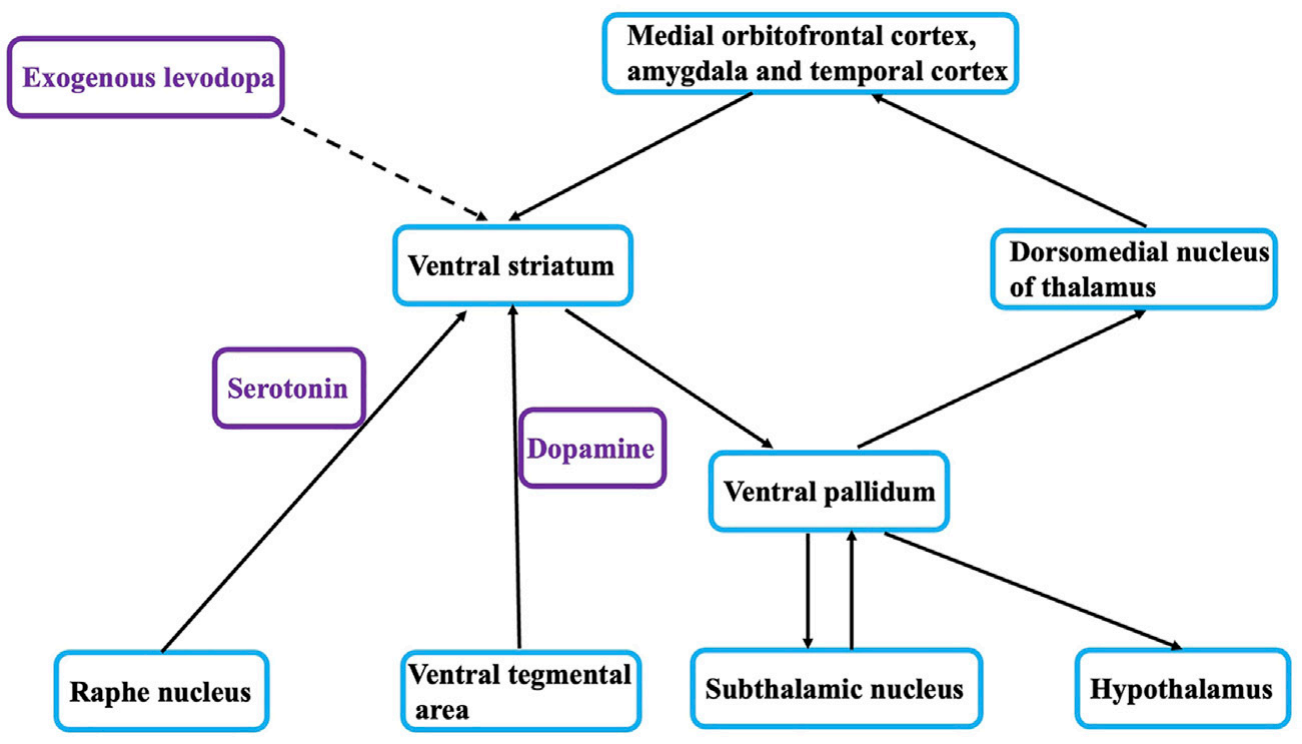
The limbic basal ganglia loop and depression.

The relationship between depression and PD remains controversial, and previous studies have helped uncover the mystery of the relationship between the two (Chen et al., 2019). α-Synuclein deposits or Lewy bodies within neurons affect brain regions in a specific order. First, the olfactory tract and lower brainstem regions are affected (stages 1 and 2), then the damage progresses upward to the lower brainstem and midbrain (stages 3 and 4), and finally it extends to the basal forebrain and cerebral cortex (stages 5 and 6). This model suggests that the serotonin-raphe nucleus of the brain stem, which is associated with emotional symptoms, is affected at stage 2, whereas the substantia nigra, which is associated with motor symptoms in Parkinson’s disease, is affected at stage 3 (Figure 1). Thus, according to this hypothesis, depression can be viewed as a prodrome of PD, involving the same pathophysiological process that later manifests as MS.

However, depression and PD can also be seen as two separate diseases, a view that can be explained by the inflammatory hypothesis that microglia are present in different areas of the brain, particularly in the substantia nigra denser (Pessoa Rocha et al., 2014). Significantly increased inflammatory responses were observed in the brain and peripheral blood of patients with PD. During the inflammatory response, activated microglia can release pro-inflammatory cytokines, reactive oxygen species, and proteins of the complement system, all of which contribute to neuronal damage and neurodegeneration (Ramirez et al., 2017; Ho, 2019). Pro-inflammatory cytokines also induce changes in release of neurotransmitters such as 5-HT, NE, and DA, similar to those in depression. Therefore, depression and PD can be caused by common pathophysiological factors, without any direct relationship between them (Lindqvist et al., 2012; Dallé et al., 2017).

In addition, some studies have suggested depression as a risk factor for PD as well as other diseases such as diabetes, tumors, dementia, and stroke (Leentjens, 2015) (Roy and Lloyd, 2012; Barlinn et al., 2015; Köhler et al., 2015; Bortolato et al., 2017); in addition, other mental and psychological problems, such as anxiety and chronic pain, are risk factors for PD (Ozturk and Kocer, 2018; Kim et al., 2020). However, in our opinion, the relationship between PD and depression is highly complex. Although PD and depression are two diseases, our clinical experience has demonstrated that PD may affect the severity and treatment effect of depression in many respects. Thus, there is a bidirectional relationship between depression and PD, and PD may also be a negative factor for depression (Ossowska and Lorenc-Koci, 2013). Exploring the relationship between PD and depression and their common pathophysiological mechanisms will contribute to the development of reasonable and alternative therapeutic strategies for DPD. Nevertheless, an increasing number of studies have shown that the efficacy of traditional antidepressants in patients with DPD is not ideal (Chen et al., 2019). Therefore, there is an urgent need to understand the neurobiological mechanisms of DPD and to clarify the treatment strategies for DPD.

CHM prescriptions can not only effectively improve depression but can also treat MS in patients with PD, thereby facilitating maintenance of patients’ mental health. TCM has played a significant role in anti-DPD in the process of treating depression and rehabilitation care, through multi-target, multi-path, multi-level, and multi-mechanism actions (Feng et al., 2021). Here, we summarize results of Case-controlled studies (CCSs) of CHM prescriptions for treating DPD and explored the anti-DPD mechanism of the active ingredients of single herbs that are predominantly used in CHM prescriptions for the treatment of depression; these data may contribute to the design of new selective and effective treatments for DPD (Zhang et al., 2014).

## Traditional Chinese Medicine in Depression in Parkinson’s Disease Treatment

Traditional single-target antidepressants have unsatisfactory therapeutic effects on DPD and may aggravate MS in PD patients (Seppi et al., 2011). TCM has a long history of treating depression, especially in Asia, where it is most common in China, Japan, and South Korea (Li et al., 2020a). With deepening of the understanding of TCM in the treatment of depression, TCM is gradually being used in the treatment of other neurological diseases with depressive symptoms, including post-stroke depression, depression in Alzheimer’s disease, depression in epilepsy, and DPD (Guo et al., 2011; Yang et al., 2021).

TCM has a unique medical theory. As a holistic medicine, TCM emphasizes the integrity of the body and the influence of the environment on homeostasis. In TCM theory, depression is caused by “qi deficiency,” which is caused by dysfunction of multiple physiological systems in the body, such as “qi stasis,” “blood stagnation,” and “phlegm obstruction.” Restoration of “vital energy” is the principle in healing depression, but disorders of other physiological systems also need to be corrected by promoting blood circulation and removing stasis, as well as regulating the liver.

To obtain international recognition and further promote CHM prescriptions, many CCSs on CHM prescriptions for the treatment of DPD have been conducted (Table 1). The YiNao JieYu decoction (YNJYD) is mainly used to treat DPD caused by kidney deficiency and liver depression. The main components of YNJYD are *Codonopsis pilosula*, *Rehmannia glutinosa Libosch*, and *Poria cocos*. Based on the treatment of 30 patients with DPD, Zhou et al. reported that YNJYD can significantly improve the depressive mood of patients with DPD. This improvement of depressive symptoms may be attributed to YNJYD increasing the levels of 5-HT, NE, and DA in the peripheral blood of DPD patients (Zhou et al., 2019). ChaiHu ShuGan (CHSGS) powder was derived from “Medical Norms,” and mainly includes *Paeoniae Rubra Radix*, *Ligusticum Chuanxiong* Hort*,* and *Bupleurum Chinense* DC. It regulates liver qi, promotes blood circulation, and relieves pain and is a representative prescription for treating liver qi stagnation syndrome. Maimaiti et al., 2012 treated 15 patients with DPD and found that all patients showed improvement in depressive symptoms after treatment (Maimaiti et al., 2012). CHSGS can affect the remodeling of hippocampal neurons and regulate neurotransmitter levels (Sun et al., 2021). In addition, CHSGS can regulate the hypothalamus‒pituitary‒adrenal (HPA) axis to play a therapeutic role in DPD. The ChaiGan JieYou decoction (CGJYD) has several effects such as clearing the liver, regulating qi, nourishing the heart, calming the mind, removing phlegm, and opening stagnation. Compared with fluoxetine, CGJYD has the advantage of significantly improving depressive symptoms and the ability to perform activities of daily living in patients with mild-to-moderate DPD; this effect may be related to an increase in brain-derived neurotrophic factor (BDNF) expression in the hippocampus (Ma et al., 2021). The BuShen HuoXue ShuGan decoction (BSHXSGD) promotes blood circulation, dispels wind, soothes the liver, and removes stagnation. BSHXSGD can not only improve MS and the ability to perform activities of daily living but also the depression symptoms in PD patients (Qin et al., 2019). Modern pharmacological studies have suggested that this effect may be achieved by dilating blood vessels, improving microcirculation, and scavenging oxygen free radicals (Qin et al., 2019). The PingChan JieYu decoction (PCJYD) nourishes the liver and kidney, dreads the liver, and relieves depression. PCJYD can improve MS and NMS (depression) in patients with PD and can reduce the dosage of levodopa and sertraline. Animal experiments have suggested that PCJYD can increase the levels of DA, 5-HT, and NE in the striatum of MPTP-PD mouse models and improve PD symptoms (Lu et al., 2010). Xiao Yao (XYD) powder, composed of *B.* DC, *Angelica sinensis*, and *Paeoniae Rubra Radix*, is produced by the “Taiping Huimin Pharmaceutical Bureau.” It harmonizes the liver and spleen, nourishes blood, strengthens the spleen, dredges the liver, and relieves depression. Case‒control studies have suggested that XYD can significantly improve the depressive symptoms of patients with PD, and its efficacy is better than that of escitalopram. Animal experiments have shown that XYD can enhance the activity of neurotransmitters and protect hippocampal neurons; additionally, it has a remodeling effect on the hippocampal synaptic structure that can reduce synaptic damage and promote the formation of new connections (Tan et al., 2012). The ZiYin HuoXue decoction (ZYHXPD) is composed mainly of *Cornus officinalis* Sieb. et Zucc, *Dioscorea opposite* Thunb, and *R. glutinosa* Libosch. It nourishes the liver and kidney, calms the liver, relieves wind, promotes blood circulation, and removes blood stasis. ZYHXPD can significantly improve the depressive symptoms and quality of life of patients with PD; however, the direct pharmacological mechanism requires further study (Zhang et al., 2013). The ZiShen PingChan decoction (ZSPCD) nourishes the liver and kidney as well as dredges and detoxifies collaterals. Compared to fluoxetine, ZSPCD can alleviate depression and sleep disorders and improve MS in PD patients (Ye et al., 2014). The JieYu YiHao decoction (JYYHD) includes *Lilium lancifolium* Thunb, *Paeoniae Rubra Radix*, and *B. chinense* DC. It soothes the liver, relieves depression, promotes qi, dispels stagnation, nourishes the heart and soothes the nerves. In a case‒control study, Wang et al. (2017) found that JYYHD may exert an anti-DPD effect by increasing the levels of NE, DA, and 5-HT.

**TABLE 1:** Characteristics of studies on TCM prescriptions in depressed PD.

TCM formulas	Compositions (pharmaceutical name)	Possible pharmacological mechanism in treating DPD	References
YiNao JieYu decoction	*Codonopsis pilosula*, *Rehmannia glutinosa* Libosch, *Poria cocos, Uncaria rhynchophylla*, *Atractylodes macrocephala* Koidz, *Angelica sinensis*, *Pinelliae ternate*, *Ligusticum chuanxiong* Hort	Increase the levels of 5-HT, NE, and DA in peripheral blood of DPD patients	Zhou et al. (2019)
ChaiHu ShuGan powder	*Paeoniae Rubra Radix*, *Ligusticum Chuanxiong* Hort, *Bupleurum Chinense* DC, Cyperus *rotundus* L, *Citrus aurantium* L, *Citrus reticulate* Blanco, *Glycyrrhiza uralensis* Fisch	Regulate the hypothalamus-pituitary-adrenal axis	Maimaiti et al. (2012)
ChaiGan JieYou decoction	*Bupleurum chinense* DC, *Citrus aurantium* L, *Paeoniae Rubra Radix*, *Glycyrrhiza uralensis* Fisch, *Triticum aestivum* L, *Ziziphus jujuba* Mill, *Polygala tenuifolia* Willd, *Acorus tatarinowii*	Increase of BDNF expression in the hippocampus	Ma et al. (2021)
BuShen HuoXue ShuGan decoction	*Pueraria montana* var. lobata, *Cyathula officinalis* Kuan, *Salvia miltiorrhiza* Bge, *Fructus Ligustri* Lucidi, *Ligusticum chuanxiong* Hort, *Gastrodia elata* Bl, *Curcuma rcenyujin* Y, H. *Chenet C.Ling, Bupleurum chinense* DC, *Chaenomeles speciose*, *Cyperus rotundus* L, *Buthus martensii* Karsch, *Polygala tenuifolia* Willd, *Yerbadetajo* Herb, *Cornus officinalis* Sieb Zucc, *Anemarrhena asphodeloides* Bge, *Glycyrrhiza uralensis* Fisch	Improve microcirculation and scavenge oxygen free radicals	Qin et al. (2019)
PingChan JieYu decoction	*Achyranthes bidentata* BI, *Citrus reticulate* Blanco, *Glycyrrhiza uralensis* Fisch, *Uncaria rhynchophylla*, *Bupleurum chinense* DC, *Fallopia multiflora*, *Ligusticum chuanxiong* Hort	Increase the levels of DA, 5-HT and NE in the striatum	Lu et al. (2010)
Xiao Yao powder	*Bupleurum chinense* DC, *Angelica sinensis*, *Paeoniae Rubra Radix*, *Atractylodes macrocephala* Koidz, *Poria cocos*, *Glycyrrhiza uralensis* Fisch, *Zingiber officinale* Rosc, *Mentha haplocalyx* Briq	Enhance the activity of neurotransmitters, protect hippocampal neurons	Tan et al. (2012)
ZiYin HuoXue decoction	*Cornus officinalis* Sieb. et Zucc, *Dioscorea opposite* Thunb and *Rehmannia glutinosa* Libosch, *Cyathula officinalis* Kuan, *Ligusticum chuanxiong* hort, *Poria cocos*, *Dendranthema morifolium*, *Fructus lycii*, *Gastrodia elata* Bl, *Alisma orientalis*, *Paeonia suffruticosa* Andr, *annia glutinosa* Libosch, *Lycium barbarum* L, *Taxillus sutchuenensis*, *Gastrodia elata* Bl, *Bombyx mori* Linnaeus, *Curcuma phaeocaulis* Valeton, *Paeoniae Rubra Radix*, *Arisaema heterophyllum* Blume	**—**	Zhang et al. (2013)
ZiShen PingChan decoction	*Gastrodia Libosch*, *Lycium barbarum* L, *Taxillus sutchuenensis*, *Paeoniae Rubra Radix*, *Gastrodia elata* Bl, *Arisaema heterophyllum* Blume, *Salvia miltiorrhiza* Bge, *Anemarrhena asphodeloides* Bge, *Lilium lancifolium* Thunb, *Buthus martensii* Karsch, *Scolopendra subspinipes* mutilans L. Koch	**—**	Ye et al. (2014)
JieYu YiHao decoction	*Lilium lancifolium* Thunb, *Paeoniae Rubra Radix*, *Bupleurum chinense* DC, *Acorus tatarinowii*, *Angelica sinensis*, *Ligusticum chuanxiong* hort, *Rosa chinensis* Jacq, *Albizia julibrissin* Durazz, *Rosa rugosa* Thunb, *Ziziphus jujuba*, *Ziziphus jujuba* Mill	Increase the levels of DA, 5-HT and NE in the brain	Wang et al. (2017)

**FIGURE 2:**
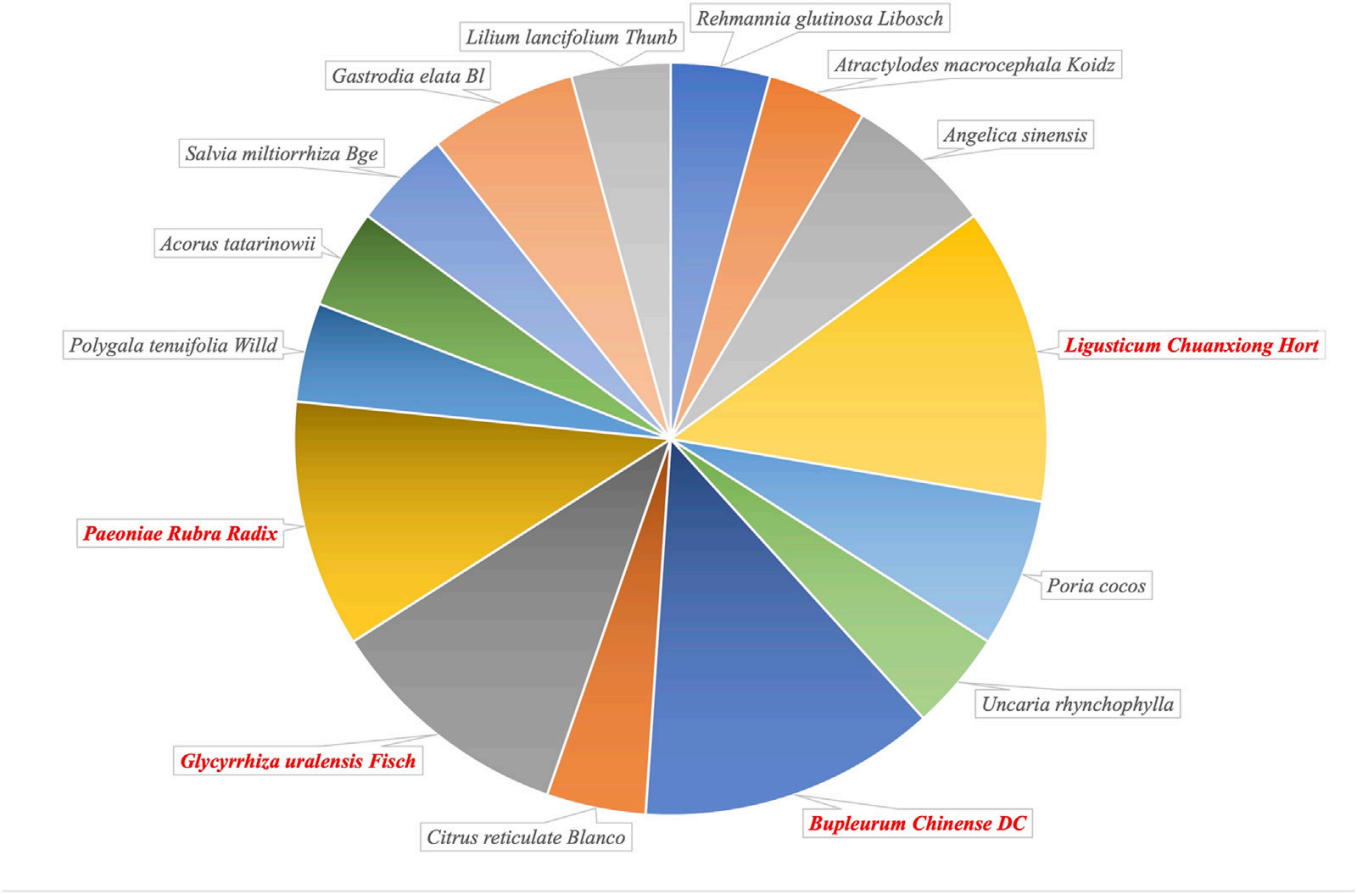
The proportion of single herbal medicines included in Traditional Chinese Medicine prescriptions confirmed clinically in the treatment of depression in Parkinson’s disease (DPD).

In summary, a variety of classical CHM prescriptions have been proven to be applicable for the treatment of DPD and demonstrated good efficacy. Notably, *L. Chuanxiong* Hort, *B. chinense* DC, *Glycyrrhiza uralensis* Fisch, and *Paeoniae Rubra Radix* are components of a number of these CHM prescriptions (Figure 2), indicating that they may play an important pharmacological role in the treatment of DPD. To explore the molecular pharmacological mechanism of the CHM prescriptions for DPD, we summarize the possible mechanism of the effective ingredients of these single herbs in treatment of depression (Table 2), to help identify new options for the effective treatment of DPD.

**TABLE 2:** Effect of antidepressant ingredient in single herbal.

Single herbal	Active ingredient	Subjects	Effects and mechanisms involved	References
*Ligusticum Chuanxiong* Hort	TMP	CUMS mice	Inhibiting the TLR4-NF-κB-NLRP3 signaling pathway, elevate the 5-HT and NE concentration in the serum and brain	Fu et al. (2019)
	TMP	CSDS mice	Increase the hippocampal BDNF/ERK expression	Jiang et al. (2015), Jiang et al. (2021)
	Ligustilide	CUMS rat	Increase levels of progesterone and allopregnanolone in the prefrontal cortex and hippocampus	Ma et al. (2021)
	Ligustilide	CUMS rat	Upregulate the hippocampal contents of tryptophan and 5-HT	Zhu et al. (2015)
	Ferulic acid	CORT mice	Repair stress caused by HPA axis dysfunction	Zeni et al. (2017)
	Ferulic acid	CUMS mice	Up-regulate BDNF expression, increase the levels of postsynaptic protein PSD95 and synapsin I	Liu et al. (2017a)
	Ferulic acid	CUMS mice	Inhibit the release of inflammatory factors and activation of microglia	Liu et al. (2017b)
	Ferulic acid	Stressed mice	Elevate serotonin and norepinephrine in mouse hippocampus and frontal cortex	Chen et al. (2015)
	Ferulic acid	Stressed mice	Increase catalase, superoxide dismutase and glutathione peroxidase in the blood, hippocampus and cerebral cortex, regulate antioxidant defense system	Lenzi et al. (2015)
	Ferulic acid	CUMS mice	Inhibit SIRT6 expression, and possibly suppress AKT/CRMP2 signaling	Li et al. (2020b)
*Bupleurum Chinense* DC	Total Saikosaponins	Stressed mice	Inhibit NE reuptake and increase serum NE level in mice	Liu et al. (2014)
	Saikosaponin D	CUMS rats	Inhibit the activation of the NF-κB signaling pathway, increase the expression of FGF2	Chao et al. (2020)
	Saikosaponin D	CUMS mice	Inhibit the expression of LPA protein in hippocampus, decreased the levels of P-P38, P-P65, P-ERK, RhoA and ROCK2	Xu et al. (2019)
	Saikosaponin D	CUMS rat	Enhance the HPA axis function	Li et al. (2017)
	Saikosaponin A	CUMS rats	Restore neuroendocrine, neuroinflammation and neurotrophic systems in the hippocampus	Chen et al. (2018)
	Saikosaponin A	CUMS rat	Up-regulate the expression level of PRRT2 and increase DA content in hippocampus	Guo et al. (2020)
*Glycyrrhiza uralensis* Fisch	Glycyrrhizic acid	Depressive patients	Inhibit extracellular cytokine activities of HMGB1 and inflammatory response	Cao et al. (2020)
	Glycyrrhizic acid	SH-SY5Y cells	Regulate autophagy, increase BDNF expression and modulate HPA activity	Yang et al. (2018)
*Paeoniae Rubra Radix*	Paeonol	LPS induced mice	Increase 5-HT and NE levels and inhibit inflammatory cytokines expression	Tao et al. (2016)
	Paeonol	CUMS mice	Promote the dendritic spines formation and regulate BDNF-Rac1/RhoA pathway	Zhu et al. (2018)
	Paeoniflorin	Reserpine-induced mice	Inhibiting inflammasome-induced pyroptosis and over-activated microglia in the depressive hippocampus	Tian et al. (2021)
	Paeoniflorin	CUMS rats	Modulate the ERK-CREB signaling pathway	Zhong et al. (2018)
	Paeoniflorin	Prenatal stress mice	Modulate the HPA axis	Li et al. (2020c)

CUMS, chronic unpredictable mild stress; CRS, chronic restraint stress; TMP, tetramethylpyrazine.

## Molecular Mechanism of Action of Traditional Chinese Medicine Herbal Antidepressants

### *L. chuanxiong* Hort

*L. chuanxiong* Hort, mainly grown in Sichuan, China, is often used to promote blood circulation and qi, dispel wind, and relieve pain. In the past, *L. chuanxiong* was considered as a qi medicine, relieving depression and pain. Several active ingredients in *L. Chuanxiong* Hort, such as tetramethylpyrazine, ligustilide, and ferulic acid, have been shown to play a role in the treatment of depression.

Tetramethylpyrazine (TMP) is a natural compound with an antidepressant-like effect but with unknown mechanisms. In chronic unpredictable mild stress (CUMS) mice, TMP inhibited Toll-like receptor 4 (TLR4) and nucleotide binding and oligomerization domain-like receptor family pyrin domain-containing 3 (NLRP3)-associated proteins in the prefrontal cortex and hippocampus. In addition, TMP can elevate 5-HT and NE concentrations in the serum and brain. In general, TMP exerts a potential antidepressant-like effect in CUMS mice by inhibiting the TLR4‒NF-κB‒NLRP3 signaling pathway in the brain (Fu et al., 2019). In chronic social defeat stress (CSDS) mice, TMP showed antidepressant effects *via* the forced swim test (FST) and tail suspension test (TST). The BDNF‒extracellular regulated protein kinase (ERK) signaling cascade plays an important role in the pathophysiology of depression. According to a previous study, BDNF levels are decreased in the hippocampus of CSDS mice, and TMP treatment increased the BDNF levels and improves depressive symptoms in these mice; these findings shed light on the development of new antidepressants with higher efficacy and fewer side effects (Jiang et al., 2015). This conclusion was further verified in a later study by Jiang et al. (2015), who suggested that TMP alleviated anxiety and depressive behaviors by increasing hippocampal BDNF/ERK expression (Jiang et al., 2021).

Ligustilide is one of the main components of *L. chuanxiong* Hort and has been reported to have antidepressant activities. In CUMS rats, ligustilide significantly increased the levels of progesterone and allopregnanolone in the prefrontal cortex and hippocampus to exert antidepressant-like effects (Ma et al., 2021). Additionally, ligustilide may upregulate the hippocampal tryptophan and 5-HT levels, causing antidepressant-like effects in rats exposed to CUMS (Zhu et al., 2015).

Ferulic acid (FA) is a natural phenolic compound found in various herbs, including *L. chuanxiong* Hort, and it has been confirmed by several studies to have excellent antidepressant effects. However, the exact pharmacological mechanisms involved are not yet well understood. Corticosterone treatment has been shown to mimic HPA axis dysregulation, implicated in the development of depression. FA has been shown to improve corticosterone-induced changes in behavior and oxidative stress, which may represent a behavioral model for the antidepressant effects of FA. FA is involved in the repair of stress caused by the HPA axis dysfunction and thus may be further investigated as a potential novel strategy to improve depression treatment (Zeni et al., 2017). FA significantly alleviated CUMS-induced depression-like behaviors in the FST of CUMS mice. FA may exert its antidepressant effects by upregulating BDNF expression and increasing the levels of postsynaptic density protein (PSD95) and synapsin I in the prefrontal cortex and hippocampus (Liu et al., 2017a). Depression is an inflammation-related mental disease. In the prefrontal cortex of a rat model of depression, interleukin (IL)-1β, IL-6, tumor necrosis factor-α (TNF-α), microglial activation, and NF-κB signaling are upregulated (Zheng et al., 2019). FA reversed this inflammatory response and improved depressive-like behaviors in both the FST and TST; this result suggested that its anti-inflammatory mechanism was involved in the antidepressant-like effects in CUMS mice (Liu et al., 2017b).

Increasing monoamine neurotransmitter levels is a classic strategy for treating depression. FA exerts a therapeutic effect on depression through elevation in the levels of 5-HT and NE, but not DA, in the mouse hippocampus and frontal cortex (Chen et al., 2015). Oxidative stress has been implicated in the pathophysiology of depression, and antioxidant therapy is one of the treatments used to improve depression. FA has been reported to increase catalase, superoxide dismutase, and glutathione peroxidase levels in the blood, hippocampus, and cerebral cortex. In animal models, FA decreased the immobility time in the TST. These data indicate that FA is a novel means to improve depression by regulating the antioxidant defense system (Lenzi et al., 2015). Sirtuin 6 (SIRT6) plays a key role in mood regulation, and knockdown of hippocampal SIRT6 can alleviate depression-like behaviors induced by CUMS in mice. Chronic treatment with FA inhibits SIRT6 expression and may suppress AKT/CRMP2 signaling, ameliorating CUMS-induced depression-like behaviors in mice (Li et al., 2020b).

### *B. chinense* DC

*B. chinense* DC is a perennial herbaceous plant. This CHM penetrates the surface, relieves heat, soothes the liver, relieves depression, and lifts Yang qi. *B. chinense* DC has antipyretic, analgesic, antiviral, anti-inflammatory, blood lipid-lowering, liver protective, and antitumor effects. Modern pharmacological studies have indicated that *B. chinense* DC contains a variety of active ingredients, among which saikosaponins have obvious antidepressant effects and may serve as a promising drug for the treatment of depression. However, the mechanism of action of saikosaponins in depression has not been fully elucidated. Liu et al. (2014) suggested that total saikosaponins could significantly shorten the immobility time of mice in the TST in a dose-dependent manner by antagonizing reserpine-induced akinesia and ptosis. Various studies have highlighted the association between NF-κB and depression-like behavior (Su et al., 2017). NF-κB plays a vital regulatory role in immune responses, including stress. Activation of NF-κB has been reported to be involved in depressive symptoms (Liu et al., 2017b). Saikosaponin D decreased NF-κB expression in CUMS rats by inhibiting the activation of the NF-κB signaling pathway related to NLRP3, which may play an important role in its effects on CUMS-induced depression-like behavior.

Moreover, saikosaponin D increases the expression of fibroblast growth factor 2 (FGF2), a growth factor related to neuronal growth and synaptic plasticity that is associated with major depressive disorder (Gupta et al., 2018) (Wu et al., 2017), both *in vivo* and *in vitro*, which provided further insight into the anti-depression mechanism of saikosaponin D (Chao et al., 2020). FGF2 is considered a new therapeutic target for depression (Lixing et al., 2018). Chao et al. (2020) Saikosaponin D also reduces LPS-induced depression-like behavior in mice. On the one hand, saikosaponin D inhibited the expression of lysophosphatidic acid (LPA) in hippocampal CA1 and CA3 regions. On the other hand, saikosaponin D significantly decreased the levels of phosphorylated (P)-P38, P-P65, P-ERK, P-I κBα, RhoA, and ROCK2 in LPS-stimulated mice as well as the expression of LPA and the degree of neuronal apoptosis in SH-SY5Y cells (Xu et al., 2019; Su et al., 2020). Moreover, treatment with saikosaponin D markedly improved the behavioral deficiency induced by CUMS. This anti-depression effect was probably achieved by downregulating serum corticosterone levels as well as alleviating the suppression of glucocorticoid receptor expression and nuclear translocation caused by CUMS; therefore, restoration of the HPA axis function may also be a therapeutic target of saikosaponin D (Li et al., 2017).

Furthermore, saikosaponin A may ameliorate depressive symptoms *via* multiple mechanisms. In a study by Chen et al. (2018), saikosaponin A was suggested to improve the regulation of the HPA axis and neuroinflammation in CUMS rats. In addition, saikosaponin A enhanced BDNF‒TrkB signaling in the hippocampus and elevated BDNF levels, resulting in antidepressant-like effects in rats. Proline-rich transmembrane protein 2 (PRRT2) is a protein that is enriched in the brain and is associated with a group of paroxysmal disorders, such as paroxysmal kinesigenic dyskinesia (Lee et al., 2012). In CUMS rats, PRRT2 expression levels were downregulated, but administration of saikosaponin A significantly counteracted this change. In addition, by upregulating PRRT2 levels, saikosaponin A increased DA levels by mediating the DA synaptic vesicle fusion and release process (Guo et al., 2020). These data provide insight into the mechanism of saikosaponin A action in the treatment of depression.

### *G. uralensis* Fisch

Use of *G. uralensis* Fisch. was first published in “Shen Nong’s Materia Medica.” It is mainly used to treat inflammatory diseases, cardiovascular and cerebrovascular diseases, and tumors, owing to the pharmacological effects mediated by its active ingredients. The main active ingredients of *G. uralensis* Fisch extract include glycyrrhiza saponin, glycyrrhizic acid (GZA), glycyrrhetinic acid, glycyrrhiza flavone, glycyrrhiza isoglycyrrhiza flavone, and glycyrrhiza polysaccharide. Of these ingredients, GZA is considered to exert definite antidepressant effects.

Proinflammatory cytokines, such as IL-1β, IL-6, and TNF-α, might play crucial roles in the pathophysiology and treatment of depression. High-mobility group box 1 (HMGB1) is a nonhistone chromatin-associated protein known for its long-term effects in initiating and amplifying neuroinflammation. Disulfide-HMGB1 induces depressive behavior in rodents by promoting the secretion of pro-inflammatory cytokines, including TNF-α (Lian et al., 2017). GZA binds directly to HMGB1, inhibits the extracellular cytokine activity of HMGB1, and alleviates the inflammatory response; thus, it may have potential in ameliorating depression (Cao et al., 2020).

Recent studies have shown that the dysregulation of autophagy is involved in the pathogenesis of DPD. In 6-hydroxydopamine (6-OHDA)- and corticosterone-induced SH-SY5Y cell models, GZA significantly increased the viability and decreased the apoptosis of SH-SY5Y cells. In addition, GZA could have a potential therapeutic effect on DPD by increasing the expression level of BDNF and by regulating HPA activity (Yang et al., 2018).

### Paeoniae rubra Radix

*Paeoniae rubra Radix* clears heat and cools the blood, disperses blood stasis, and relieves pain. Recent pharmacological studies have shown that *Paeoniae rubra Radix* contains terpenoids, flavonoids and volatile oils; these ingredients have various pharmacological effects, such as liver protection, antitumor, neuroprotection, heart protection, anti-oxidant, and anti-endotoxin activities and so on (Genfa et al., 2005; Zhang et al., 2011; Jiao et al., 2021).

Emerging studies have shown that paeoniflorin and paeonol, the two main active components of *Paeoniae rubra Radix*, may play a role in the treatment of depression through various mechanisms. Paeonol, the principal active component of *Paeoniae Rubra Radix*, has been extensively studied for its antioxidant, anti-inflammatory, and anti-atherosclerotic effects. In mice with LPS-induced depression-like behavior, paeonol showed significant antidepressant effects. To further explore the antidepressant pharmacological mechanism of paeonol, Tao et al. (2016) investigated LPS-induced depressive mice and found that paeonol increased 5-HT and NE levels in the hippocampus and reduced the levels of inflammatory cytokines levels (IL-6 and TNF-α level) in rat serum, suggesting that paeonol is an active component with therapeutic potential against DPD (Tao et al., 2016). In addition, paeonol attenuated CUMS-induced depression-like behaviors accompanied by hippocampal neuronal morphological alterations (Zhu et al., 2018).

Activation of cofilin1 is essential for remolding dendritic spines. Paeonol treatment inhibits cofilin1 activation and increases the dendritic length and density of dendritic spines in the hippocampal CA1; moreover, it upregulates BDNF levels and downregulates Rac1/RhoA levels, thus mediating antidepressant effects (Zhu et al., 2018).

Paeoniflorin, an organic compound extracted from the roots of *Paeoniae rubra Radix*, has been shown to exert antidepressant effects. Gasdermin D (GSDMD)-mediated pyroptosis in activated microglia is involved in the pathogenesis of depression. Tian et al. demonstrated that paeoniflorin inhibits the enhanced expression of GSDMD and prevents LPS- and adenosine triphosphate-induced pyroptosis in murine N9 microglia *in vitro*. Therefore, paeoniflorin may exert antidepressant effects by alleviating neuroinflammation and inhibiting caspase-11-dependent pyroptosis signaling (Tian et al., 2021).

The ERK signaling pathway plays a pivotal role in the regulation of depression (Zhu et al., 2019). In a study, paeoniflorin treatment markedly increased the mRNA levels of *ERK1*, *ERK2*, and *CREB* in the hippocampus and decreased the degree of neuronal damage in the hippocampus of rat models. Therefore, paeoniflorin may exert a neuroprotective effect that is modulated by the ERK‒CREB signaling pathway in rats with CUMS-induced hippocampal damage (Zhong et al., 2018).

Furthermore, paeoniflorin exerts antidepressant effects by modulating the HPA axis. Paeoniflorin can reduce serum corticosterone, adrenocorticotropin, and hippocampal glutamate levels in prenatally stressed mice; additionally, it markedly increases neurogranin levels in the hippocampal CA3 region of depressed mice (Li et al., 2020c).

## Perspective and Conclusion

In TCM theory, DPD is a combination of “*Chan Zheng*” and “*Yu Zheng.*” The clinical efficacy of conventional antidepressants, such as 5-HT-reuptake inhibitors, in DPD has been increasingly questioned. These drugs are not ideal for improving depression symptoms and have shown no positive effects on PD MS. The therapeutic effects of CHM prescriptions are often non-specific and can be neither studied nor explained by the principles of specific therapies. A CHM prescription is a complex system used to treat the complex human systems and thus has the advantage of comprehensively considering multiple targets. Recent case‒control studies have applied CHM prescriptions for the treatment of DPD and have achieved good therapeutic effects, suggesting that CHM prescriptions have broad prospects for the treatment of NMS in patients with PD. Importantly, these prescriptions can also improve MS in patients with PD while treating NMS.

**FIGURE 3:**
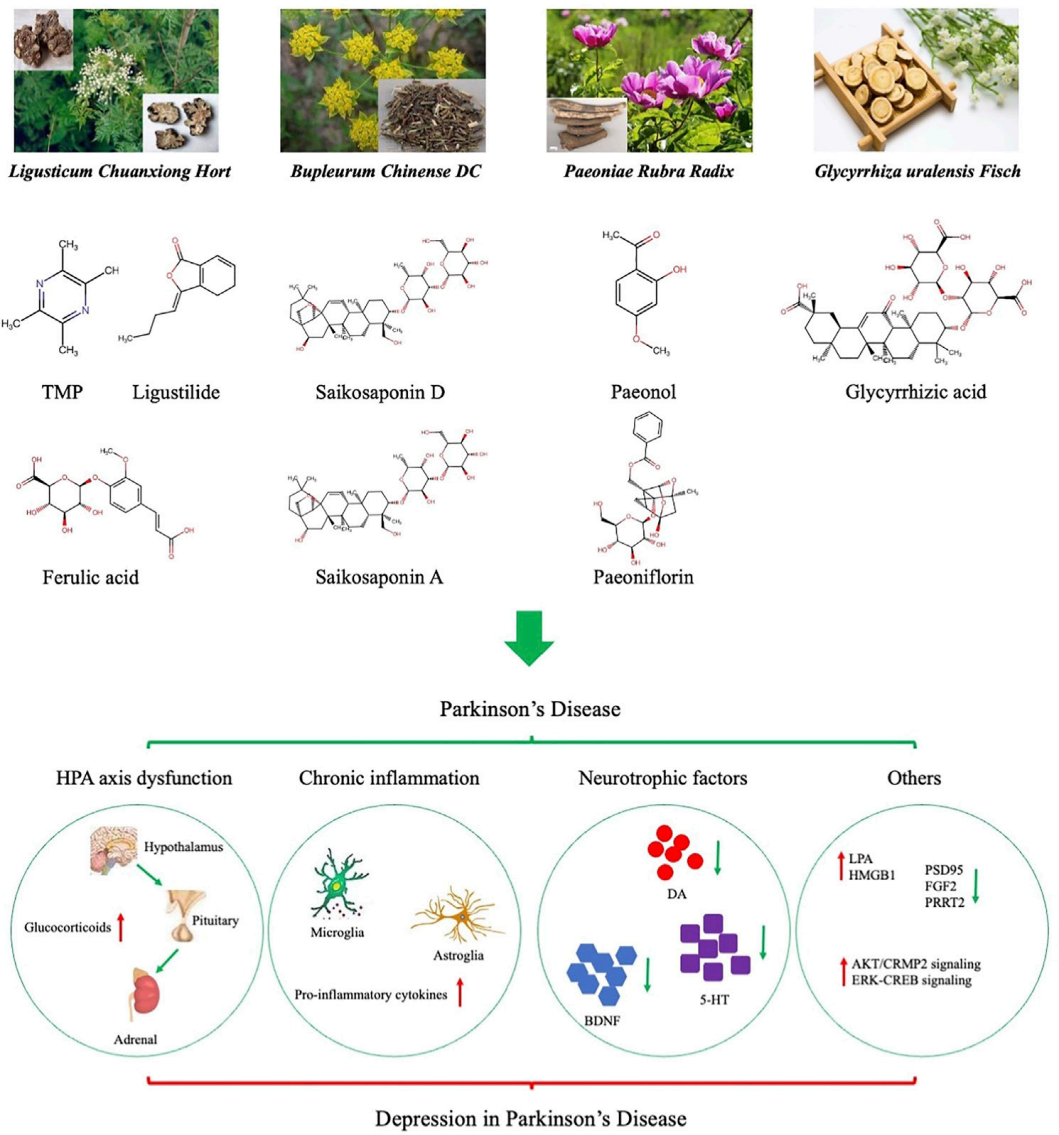
Active ingredients of single herbal medicines used in treating depression in Parkinson’s disease (DPD). Dysfunction of the hypothalamic‒pituitary‒adrenal (HPA), activation of chronic inflammation, decrease of neurotrophic factors, and other mechanisms play a central role in the pathophysiology of DPD.

Pharmacological studies of the active ingredients in single herbs are indispensable for promoting the clinical application of CHM prescriptions. We reviewed the pharmacological mechanisms of the active ingredients of the predominant single herbs found in CHM prescriptions for depression. Basic pharmacological studies have shown that these active ingredients improve depression symptoms through a variety of mechanisms, including regulation of HPA axis activity, inhibition of the inflammatory response of the central nervous system, and promotion of the release of BDNF (Figure 3). In addition, some active ingredients contribute to α-synuclein elimination, which is the core pathological change in PD. For example, FA could prevent the oligomerization of α-synuclein and reduce synaptic toxicity (Takahashi et al., 2015), TMP analogs promote the clearance of α-synuclein by enhancing proteasome activity in PD models (Zhou et al., 2019), and paeoniflorin promotes the degradation of α-synuclein by enhancing both autophagy and ubiquitin–proteasome pathways (Cai et al., 2015). These studies suggest that the active ingredients contained in CHM prescriptions provide a pharmacological basis for the treatment of NMS in patients with PD. Considering the possibility of improving MS in PD patients simultaneously, these CHM prescriptions may be the first choice for treatment of DPD.

However, there are some limitations to the clinical application of CHM prescriptions in the treatment of DPD. First, CHM prescriptions include a variety of herbs, and the pharmacokinetic changes of these herbs in the human body need to be studied further to elucidate their synergistic mechanisms. Second, the active ingredients in single herbs have been proven to have a therapeutic effect on depression through multiple mechanisms in animal experiments; however, clinical trials in humans are lacking. Finally, although the pathogenesis of DPD shares some similarities with that of depression, the pathological mechanisms of DPD have not been fully elucidated. Further study of the exact pathogenesis of DPD is necessary to promote the application of CHM prescriptions in DPD treatment.

In conclusion, although most studies to date have suggested that DPD shares a pathogenesis with depression, current research has only uncovered a corner of the mystery of DPD, and the pathological mechanism of DPD needs to be explored further. In particular, the relationship between DPD and PD needs to be elaborated. Numerous small case‒control studies have suggested that CHM prescriptions have achieved good results in the treatment of DPD, as well as in improvement of MS in patients with PD. However, the synergistic mechanisms of various herbs in T CHM prescriptions need to be explored further. The active ingredients in herbal medicines have been shown to improve depressive symptoms in animal models *via* multiple mechanisms; these findings provide impetus for the further exploration of the treatment of DPD using CHM. Elucidation of the exact pathogenesis of DPD and further clinical studies on the treatment of DPD with CHM prescriptions may promote the wide application of CHM in the future.
